# A technique to aid the insertion of distal locking screws

**DOI:** 10.1308/003588412X13373405385214h

**Published:** 2012-07

**Authors:** PR Middleton, L Ng, A Humphrey

**Affiliations:** ^1^County Durham and Darlington NHS Foundation Trust,UK; ^2^Newcastle upon Tyne Hospitals NHS Foundation Trust,UK

## BACKGROUND

The insertion of distal locking screws on an intramedullary nail can be problematic as there is no hardware such as a jig to aid insertion. Newly developed computer aided techniques are available but the cost can be prohibitive. Insertion of distal screws usually relies wholly on image intensifier guided positioning. This can be time consuming and requires many x-rays to be taken in order to create the ‘perfect circles’ on the image intensifier that demonstrate satisfactory alignment. We propose a technique that decreases insertion time and requires fewer image intensifier images to be taken while inserting a distal locking screw.

## TECHNIQUE

Pass a drill guide through the jig used for inserting proximal screws. Leave the drill guide in position against the limb. This can now be used to align the C-arm. Position the C-arm distally and adjust its position until it is parallel to the drill guide in its orbital and swivel axis. This will give near perfect alignment to the distal locking holes. Finer adjustments may be needed under image intensifier guidance to gain the final position. Once satisfactory alignment is achieved, insert the distal screws as usual.

## DISCUSSION

This simple technique is effective, saves time and reduces radiation exposure to both the patient and surgeon. This technique is free as it uses standard equipment that comes with the nail.

